# Aging in Rural Communities: Advocacy Does Matter

**DOI:** 10.1093/geroni/igaa057.1858

**Published:** 2020-12-16

**Authors:** Lisa Wiese, Lyn Holley, M Aaron Guest

## Abstract

Disparities in health care information and access often experienced by rural residents exacerbate health risks of older adults. The purpose of this symposium is to highlight efforts in varied rural settings to develop new information that contributes to identifying, assessing, and addressing the causes of these disparities, thus informing and strengthening advocacy and intervention. Mi and Ma address an unmet need for the description of challenges related to the extensive migration of Chinese rural workers and their families (46 million increase since 2011) that has exacerbated disparity of Social Security access. They describe barriers to establishing eligibility of these newly urban families for social services as they change from rural farmers to urban householders. Asman and Sisofo discuss barriers to working successfully with an alliance of diverse city, county, and state organizations to meet perceived needs of three categories of rural older adults: 1)active but denies aging, 2)adapting to aging and achieving satisfaction; and 3)dependent aging. Using a novel measure to determine true needs of their rural older communities resulted in benchmarks, which the alliance used in creating their strategic plan. Ford and colleagues tested a church-based program in a rural African American setting to facilitate chronic disease management. They tested the efficacy of utilizing online resources in underserved settings. Wiese and Williams investigated stakeholder perceptions/knowledge regarding dementia at six diverse rural sites. Although 71% of participants believed heart disease and poor diet increased dementia risk and were willing to be screened, only 15% had been tested for memory loss.

